# Erythropoietin: New Directions for the Nervous System

**DOI:** 10.3390/ijms130911102

**Published:** 2012-09-06

**Authors:** Kenneth Maiese, Zhao Zhong Chong, Yan Chen Shang, Shaohui Wang

**Affiliations:** 1 Laboratory of Cellular and Molecular Signaling, Cancer Center, F 1220, New Jersey Health Sciences University, 205 South Orange Avenue, Newark, NJ 07101, USA; E-Mails: zzchong@yahoo.com (Z.Z.C.); ycshang2000@yahoo.com (Y.C.S.); wsh2078@gmail.com (S.W.); 2 Cancer Institute of New Jersey, New Brunswick, New Jersey 08901, USA; 3 New Jersey Health Sciences University, Newark, New Jersey 07101, USA

**Keywords:** Akt, Alzheimer’s disease, amyotrophic lateral sclerosis, apoptosis, cancer, erythropoietin, mTOR, oxidative stress, Parkinson’s disease, PI 3-K, Wnt

## Abstract

New treatment strategies with erythropoietin (EPO) offer exciting opportunities to prevent the onset and progression of neurodegenerative disorders that currently lack effective therapy and can progress to devastating disability in patients. EPO and its receptor are present in multiple systems of the body and can impact disease progression in the nervous, vascular, and immune systems that ultimately affect disorders such as Alzheimer’s disease, Parkinson’s disease, retinal injury, stroke, and demyelinating disease. EPO relies upon *wingless* signaling with Wnt1 and an intimate relationship with the pathways of phosphoinositide 3-kinase (PI 3-K), protein kinase B (Akt), and mammalian target of rapamycin (mTOR). Modulation of these pathways by EPO can govern the apoptotic cascade to control β-catenin, glycogen synthase kinase-3β, mitochondrial permeability, cytochrome c release, and caspase activation. Yet, EPO and each of these downstream pathways require precise biological modulation to avert complications associated with the vascular system, tumorigenesis, and progression of nervous system disorders. Further understanding of the intimate and complex relationship of EPO and the signaling pathways of Wnt, PI 3-K, Akt, and mTOR are critical for the effective clinical translation of these cell pathways into robust treatments for neurodegenerative disorders.

## 1. Introduction

The concept of biological agents functioning as hormones may have had its early origins with Ernest Starling when he introduced the term to the Royal College of Surgeons in 1905 [1]. Starling was discussing the potential existence of agents in the blood that could stimulate organs in the body and chose the term “hormone” that was derived from the Greek term meaning to “excite” or “arouse” [2]. During this period, Carnot and Deflandre were investigating the agent “hemopoietine” [3]. They removed plasma following a bleeding stimulus in rabbits and demonstrated that injecting this plasma into untreated animals would promote the development of immature red blood cells. Other work confirmed the findings of Carnot and Deflandre to show that plasma obtained by bleeding animals acted as a stimulus to produce new red blood cells in untreated animals [4–6]. As “hemopoietine” became known as erythropoietin (EPO), studies later demonstrated that loss of oxygen tension in one parabiotic rat would lead to reticulocytosis in the normoxic partner [7]. With the subsequent purification of human EPO, the EPO gene was cloned and approval was obtained for the clinical use of recombinant EPO [8,9].

## 2. EPO Structure and Expression

### 2.1. Molecular Structure of EPO

The EPO gene is a single copy in a 5.4 kb region of the genomic DNA on chromosome 7 and leads to the initial encoding of a polypeptide chain containing 193 amino acids [10,11]. EPO is subsequently processed into a 166 amino acid peptide with the cleavage of a 27 amino acid hydrophobic secretory leader at the amino-terminal [12]. In position 166, a carboxy-terminal arginine is deleted in the mature human and recombinant human EPO (rhEPO) leading to a mature protein of 165 amino acids with a molecular weight of 30.4 kDa [13,14]. EPO has four glycosylated chains that include three *N*-linked and one *O*-linked acidic oligosaccharide side chains. The *O*-linked sugar chain is composed of Gal-GalNAc and sialic acids and *O*-linked glycosylation occurs at serine^126^. The three *N*-glycan chains consist of a tetra-antennary structure with or without *N*-acetyllactosamine repeating units and *N*-linked glycosylation occur at aspartate^24^, aspartate^38^, and aspartate^83^. The production, secretion, longevity, and function of EPO depend upon the *N*- and *O*-linked chains [15]. For example, replacement of asparagine^38^ and asparagine^83^ by glutamate or serine^126^ by glycine can impair the production and secretion of EPO [16]. The oligosaccharides in EPO may protect against oxygen radical activity [17] and the *N*-glycosylated chains are believed to contribute to the thermal stability of EPO [18]. Biological activity for EPO depends upon two disulfide bonds formed between cysteine^7^ and cysteine^160^ as well as between cysteine^29^ and cysteine^33^ [19]. Alkylation of the sulfhydryl groups results in irreversible loss of the biological activity of EPO.

### 2.2. Tissue Expression of EPO

At the cellular level, EPO expression is regulated by oxygen tension rather than through the concentration of red blood cells [2,15]. Hypoxia-dependent expression of EPO and the EPO receptor (EPOR) are modulated through hypoxia-inducible factor 1 (HIF-1) [10,11,20,21] that also may have independent pathways of cytoprotection [22–24]. Gene transcription of EPO and EPOR directly results from the activation of HIF-1 and is controlled through the transcription enhancer region in the 3′-flanking region of the EPO gene that binds to HIF-1 [10,11]. However, other stimuli that do not involve hypoxia also can affect the expression of EPO and its receptor. During free radical exposure, EPO and the EPOR are present in cerebral endothelial cells (ECs) and remain biologically active to offer cellular protection against apoptotic cell death [25]. Free radical exposure in neurons also leads to increased HIF-1 expression and subsequent increase in EPO expression [20]. EPO and the EPOR are expressed in experimental models of Alzheimer’s disease during aging [26] and in renal tubular cells during high glucose-induced oxidative stress [27]. Serum EPO levels are significantly increased during systemic infections such as malaria [28,29]. Loss of endogenous anti-oxidants such as selenium also can promote and increase EPO expression [30]. Anemic stress, insulin release, and several cytokines, including insulin-like growth factor, tumor necrosis factor-α (TNF-α) [31], interleukin-1β (IL-1β), and interleukin-6 (IL-6) also can result in increased expression of EPO and the EPOR [11,32,33]. Other cellular changes, such as hypoglycemia, cadmium exposure, raised intracellular calcium, or strong neuronal depolarizations also can alter the expression of EPO [15,34,35].

Although EPO is produced and secreted in several organs throughout the body that include the brain, liver, and uterus [33,34,36–38] and is detected in the breath of individuals [39], the kidney peritubular interstitial cells are the principle site for the production and secretion of EPO [38,40]. EPO also can provide protection for renal cells during toxic insults [41,42]. In the liver, EPO has been shown to protect against ischemic-reperfusion injury [43], but excessive over-expression of EPO can lessen the beneficial effects of EPO [44]. EPO also has been shown to have increased expression in amniotic fluid during fetal hypoxia, preeclampsia, and during diabetic pregnancies [45]. This intrauterine increase in EPO may be neuroprotective since EPO application can lessen retinal injury during intrauterine inflammation [46].

Current work has demonstrated that EPO is expressed throughout the body and may affect multiple biological functions even though EPO is presently approved by the Food and Drug administration for the purpose of treating anemia. For example, in the nervous system, EPO can be produced and secreted in neurons of the hippocampus, cortex, internal capsule, midbrain, and nervous system tumors [13,14,47]. EPO also is present in myoblasts, peripheral ECs, cardiomyocytes, and insulin-producing cells [2,10,48,49]. Yet, it is important to note that the expression of EPO and the EPOR may lead to variable biological outcomes that can be beneficial for nervous system disorders, but also may promote detrimental outcomes such as aggressive tumor growth and decreased overall survival [50]. For these reasons, knowledge of the underling cellular pathways governed by EPO is crucial for future translation of safe and effective therapeutic strategies for neurodegenerative disorders.

## 3. EPO and Cytoprotection in the Nervous System

### 3.1. EPO in the Central and Peripheral Nervous Systems

EPO plays a significant role in both the developing nervous system and the mature nervous system. In murine models, EPO gene expression is present by embryonic day ten in the brain at comparable levels found in the bone marrow and spleen [51]. The EPOR also is expressed in the human peripheral nervous system on myelin sheaths of radicular nerves [52]. EPO production in the brain is elevated during gestation, but is reduced following maturation to be controlled by the need to maintain oxygen homeostasis for tissues [2,53]. Decreased oxygen tension increases EPO production in both peripheral organs and the brain [34,54].

### 3.2. EPO and Neuronal, Vascular, and Related Cardiac Protection

The presence of EPO and its receptor in the neurovascular system has generated an immense amount of interest to target EPO and its downstream pathways for novel therapeutic strategies against neurodegenerative disorders. EPO can protect neurons from oxidative stress [55–59], spinal cord ischemia [60], retinal disease [36,46,61,62], stroke [49,63], and demyelinating disease [64]. EPO also can promote bone formation in spinal fusion models [65], modulate vascular dilatation [66], may reduce cerebral aneurysm formation [67] and prevent endothelial cell injury [25–76], protect non-neuronal cells [37,77–80], block disability during infection [28,29,46,81], limit β-amyloid (Aβ) degeneration [26,79,82,83], and may foster memory function [26]. In related systems that directly affect central nervous system function such as the cardiac system, EPO can prevent cardiac injury during chemotherapy [84], improve cardiac contractile function [85], limit cardiac failure through the reduction of inflammation, fibrosis, and oxidative stress [86], and reduce nitrosative stress [87]. These benefits of EPO in the cardiovascular system should correlate with improved cerebral perfusion during cardiac injury. It should be noted that not in all cases EPO may be beneficial, since some studies suggest no improvement for cardiac protection following cardiac ischemia and sometimes the potential for adverse effects [88].

### 3.3. EPO and Neurodegenerative Disorders

During chronic neurodegenerative disorders such as cognitive loss and Alzheimer’s disease, EPO may prevent cell toxicity, reduce β-amyloid burden, and lead to improvements in memory [26,79,82,83,89,90]. In models of Parkinson’s disease, EPO represses expression of the pro-apoptotic protein p53 up-regulated modulator of apoptosis (PUMA) [91] and prevents l-3,4-dihydroxyphenylalanine (l-DOPA) toxicity through reductions in caspase 3 activity [57]. In experimental autoimmune encephalomyelitis (EAE), EPO can suppress EAE that is associated with an increase in the number of astrocytes expressing tissue inhibitor of metalloproteases [64] and prevent demyelination in combination with methotrexate administration [92]. In some models of amyotrophic lateral sclerosis, EPO may preserve motor neurons, reduce inflammation [93,94], and prevent aggregation of mutant copper/zinc-binding superoxide dismutase [95], but EPO in amyotrophic lateral sclerosis models may not prolong life span [96]. EPO also may be associated with the treatment of depression and has been shown in animal models to have increased expression during electroconvulsive therapy and reduce depressive behavior [97]. In studies with seizures, EPO reduced seizure duration, protected against hippocampal cell loss, and decreased hippocampal neuronal cell apoptosis [98].

## 4. EPO, Oxidative Stress, and Apoptosis

### 4.1. EPO and Oxidative Stress

Oxidative stress impacts every system of the body and can lead to cell death in the vasculature system [73,99–103], the immune system [104–106], the cardiac system [84,107–110], and the brain [111–119]. Oxidative stress also may be a contributing factor to the complications of diabetes mellitus [109,120–125] and cerebral cognitive loss [126,127]. Oxidative stress is the result of the generation of reactive oxygen species (ROS) that are formed through superoxide free radicals, hydrogen peroxide, singlet oxygen, nitric oxide (NO), and peroxynitrite [128–130]. ROS are usually maintained at non-toxic levels by endogenous antioxidant systems that include superoxide dismutase, catalase, glutathione peroxidase, and vitamins C, D, E, and K [131–133]. ROS if not controlled by antioxidant systems can affect mitochondrial function, DNA integrity, and protein folding that result in cell death [121,123,129,134–138]. Studies have associated oxygen free radical production with DNA damage in diabetic patients [139,140], mitochondrial injury and aging mechanisms [137,141,142], and nutritional impairment [143].

EPO has been demonstrated to directly limit cell injury and ROS generation during oxidative stress. EPO can block the generation of ROS [27], may prevent oxidative stress at high altitudes [144], and is cytoprotective against oxidative stress that is stimulated by tumor necrosis factor-α (TNF-α) [73]. EPO also can limit oxidative stress injury during cisplatinum administration [42,145] and in models of Parkinson’s disease [57]. EPO can preserve cellular integrity in neurons [35,55,82,146,147], vascular cells [25,68–73,76,148], and inflammatory cells of the nervous system [37,77–79,149] during oxidant stress mediated injury.

### 4.2. EPO and Apoptotic Injury

Oxidative stress can lead to cell injury through pathways of programmed cell death that involve apoptosis. Apoptosis consists of the cleavage of genomic DNA that usually is not a reversible process [68,91,150]. Enzymes responsible for DNA degradation include the acidic, cation independent endonuclease (DNase II), cyclophilins, and the 97 kDa magnesium-dependent endonuclease [151–154]. Three separate endonuclease activities also exist in the nervous system, including a constitutive acidic cation-independent endonuclease, a constitutive calcium/magnesium-dependent endonuclease, and an inducible magnesium dependent endonuclease [2,155]. Apoptosis also has an early phase that involves the exposure of membrane phosphatidylserine (PS) residues [123]. The early phase can label injured cells with membrane PS residues and alert inflammatory cells to engulf and remove these injured cells [156,157]. For this to occur such as during periods of oxidative stress, inflammatory cells increase their expression of the phosphatidylserine receptor (PSR) on the membrane surface [77,158]. As a possible therapeutic strategy, membrane PS externalization can be reversed and blockade of the PSR receptor can limit activation and proliferation of inflammatory cells during apoptosis [55,159] to prevent the engulfment of functional cells that may consequently be labeled with membrane PS exposure [160,161].

Activation of caspases occurs during apoptosis [89,162,163]. In the extrinsic pathway, the intracellular death domain of death receptors, such as the tumor necrosis family (TNF) superfamily, Fas/CD95/Apo-1, can bind to extracellular ligands and lead to an intracellular death-inducing signaling complex following recruitment of adaptor molecules, such as the Fas associated death domain (FADD). FADD recruits caspase 8 and 10 through its DED domain to result in the activation of caspase 8 and 10. Caspase 8 can result in caspase 3 activation. Caspase 8 also can cleave BH3-only protein Bid, a pro-apoptotic member of the Bcl-2 family and result in truncated Bid (tBid) that promotes cytochrome c release through Bax resulting in the subsequent activation of executioner caspases. For intrinsic caspase pathway, mitochondrial membrane depolarization releases cytochrome c and activates caspase 9 and caspase 3. This is regulated by the Bcl-2 subfamily BH3-only proteins including Bid, Bad, Bim, Bmf, Puma, and Noxa, which are normally located in cellular compartments other than mitochondria. The translocation of these proteins to mitochondria associate with Bax, a multiple Bcl-2 homology domain containing protein, to promote permeabilization of the outer mitochondrial membrane and the release of cytochrome c. Cytochrome c binds to apoptotic protease activating factor-1 (Apaf-1) that consists of three different domains that include CARD, repeats of tryptophan and aspartate residues (WD-40 repeats), and a nucleotide-binding domain CED-4. Binding of cytochrome c to Apaf-1 results in the removal of the WD-40 domain, masking the CED-4 and CARDs, and leads to the oligomerization of Apaf-1. The oligomerization of Apaf-1 promotes the allosteric activation of caspase 9 by forming the Apaf-1 apoptosome. Caspase 9 can subsequently activate caspase 3 as well as caspase 1 through the intermediary caspase 8. Caspase 1 and caspase 3 activation result in DNA fragmentation and membrane PS exposure [164–166].

EPO can modulate a number of components in the apoptotic cascade to avert cell death. EPO has been shown to prevent mitochondrial depolarization and the subsequent release of cytochrome c [56,68,69,167,168]. EPO can control mitochondrial signaling through Bad, Bax, Puma [27,55,58,76,79,84,91]. EPO also blocks Apaf-1 activation [25,78] and prevents the early activation of several caspases such as caspase 1, caspase 3, and caspase 9 [25,27,44,55,57,59,72,79,87,169,170].

## 5. EPO and Novel Neuroprotective Pathways

### 5.1. EPO and Wingless

Wnt proteins are cysteine-rich glycosylated proteins derived from the *Drosophila Wingless* (*Wg*) and the mouse *Int-1* genes that oversee multiple biological functions such as stem cell development, vascular growth, maturation of the nervous system, neurodegeneration, and cognition [171–174]. Wnt signaling has been linked to frontotemporal dementia [175], the transcriptional regulation of neurodegenerative pathways [176], and late onset Alzheimer’s disease [177]. Some studies suggest that activation of the Wnt pathway may provide a therapeutic target for Alzheimer’s disease [178]. The wingless family member Wnt1 can have increased expression during injury to the neurovascular system. Wnt1 expression is increased during cortical injury [179], upon endothelial cell [68,71] exposure to elevated glucose [68,71], during spinal cord injury [172], in reactive central nervous system astrocytes [180], and during vascular cell aging [181]. Wnt1 has been shown to reduce cerebral infarct size and improve neurological function following the onset of cerebral ischemia in rats [179]. Wnt1 also prevents protects against cell loss in dopaminergic neurons in models of Parkinson’s disease [182,183], limits vascular injury during experimental diabetes [68,71], maintains microglial cell survival during Aβ exposure [79,106,184]. Loss of Wnt1 signaling can result in apoptosis [79,185–187].

**Figure 1:**
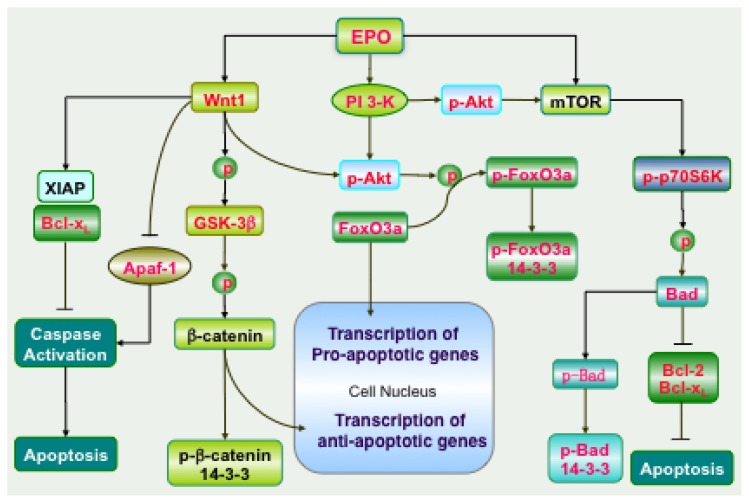
Erythropoietin (EPO) employs novel signaling pathways to prevent apoptotic cell death. EPO can stimulate the phosphoinositide-3-kinase (PI 3-K) and subsequently lead to the activation of Akt. Akt can phosphorylate the forkhead transcription factor FoxO3a to prevent its nuclear translocation and transcription of “pro-apoptotic” genes. EPO through Wnt1 phosphorylates Akt and glycogen synthase kinase-3β (GSK-3β) to prevent β-catenin phosphorylation by GSK-3β and promote the nuclear translocation of β-catenin to increase transcription of “anti-apoptotic genes”. Phosphorylated FoxO3a and β-catenin are recruited and bound by cytoplasmic docking protein 14-3-3. In addition, EPO also integrates Wnt1 to regulate the expression of X-linked inhibitor of apoptosis protein (XIAP), anti-apoptotic protein Bcl-x_L_, and apoptotic protease activating factor-1 (Apaf-1). These processes prevent caspase activation and the induction of apoptosis. Mammalian target of rapamycin (mTOR) is another target for EPO to prevent apoptosis. Following activation of mTOR, p70 ribosomal S6 kinase (p70S6K) is phosphorylated and activated. The activated p70S6K increases the expression of Bcl-2/Bcl-x_L_, phosphorylates Bad, and results in the dissociation of Bad with Bcl-2/Bcl-x_L_. This leads to an increase in the binding of Bad to the protein 14-3-3 and more available Bcl-2/Bcl-x_L_ to prevent apoptosis.

EPO uses Wnt1 and its signaling pathways such as β-catenin to prevent apoptotic cell injury. In models of experimental diabetes, EPO preserves brain EC integrity that is necessary for protection of the neurovascular unit through Wnt1, since administration of anti-Wnt1 neutralizing antibodies or gene silencing of Wnt1 block EPO protection (Figure 1) [68,71]. EPO also uses Wnt1 to maintain and translocate β-catenin to the cell nucleus to initiate “anti-apoptotic” pathways and also prevent activation of the “pro-apoptotic” pathways of glycogen synthase kinase-3β (GSK-3β) [68]. EPO also has been shown to improve Wnt family signaling in mesenchymal stem cells and increase their resistance against a neurotoxic environment [188]. Wnt1 can modulate Apaf-1 and X-linked inhibitor of apoptosis protein (XIAP) through EPO to maintain microglial cell survival during oxygen-glucose deprivation (OGD) [78]. In addition, the potential protective capacity of EPO and Wnt1 during Alzheimer’s disease may be linked to the ability of EPO and Wnt1 to govern Bad, Bcl-x_L_, and caspase activity and increase microglial cell survival during Aβ toxicity [79].

### 5.2. EPO, PI 3-K, and Akt

Although outside of the traditional *wingless* canonical and non-canonical signaling, Wnt pathways have recently been shown to rely upon pathways such as phosphoinositide 3-kinase (PI 3-K) and protein kinase B (Akt) [68,178,179,184,189–194]. PI 3-K, and Akt can prevent cell injury and the onset of apoptosis in multiple systems of the body. PI 3-K and Akt can promote cellular proliferation and block apoptotic injury either alone or through pathways that involve EPO. In regards to the nervous system, activation of PI 3-K and Akt can promote endothelial survival [66,68,69,72,100,101,195,196], prevent cell injury in inflammatory cells [77,105,165,197–200], and block neuronal injury [58,157,179,201–205]. Akt also can limit apoptosis through the phosphorylation of FoxO proteins [206–210]. For example, Akt phosphorylates the residue of serine^253^ of FoxO3a resulting in its export from the nucleus to the cytoplasm and blocking FoxO3a from activating apoptotic genes. One caveat for the PI 3-K and Akt pathways are their ability to promote cell growth that sometimes may lead to tumorigenesis if not kept in check. Under these conditions, removing PI 3-K and Akt activity can increase radiosensitivity of tumors [211] and limit the growth of tumors in the nervous system such as medulloblastomas [189].

PI 3-K phosphorylates membrane lipids and mediates the transition of Akt from the cytosol to the plasma membrane. Subsequently, Akt is phosphorylated on the residues of serine^473^ and threonine^308^ by phosphoinositide dependent kinase (PDK) PDK1 and PDK2. EPO employs these pathways to phosphorylate Akt at serine^473^ and lead to its activation (Figure 1). As an example, EPO requires Akt for the mobilization of multipotent stromal cells [212]. EPO also can protect dorsal root ganglion neurons in animal models of diabetes mellitus with streptozotocin through pathways that activate Akt [213]. EPO relies upon Akt activation in pathways that require sirtuins to maintain cerebral vascular cell survival during oxidative stress [72]. EPO utilizes Akt for the post-translational phosphorylation of FoxO proteins to maintain FoxO transcription factors in the cytoplasm by association with 14-3-3 proteins and prevent the transcription of “pro-apoptotic” genes [70]. In retinal cells, EPO is cytoprotective against the stress of glyoxal-advanced glycation end products (AGEs) through activation of Akt [58] and EPO may rely upon Akt during retinal detachment [214]. During several toxic cellular environments, Akt appears to be necessary for EPO to foster protection such as during Aβ exposure [78,79,83,90], hypoxia [69,215], and oxidative stress [55,216,217].

### 5.3. EPO and mTOR

Both PI 3-K and Akt have significant roles in modulating the activity of the mammalian target of rapamycin (mTOR) to control cell growth and proliferation [99,107]. mTOR is a 289-kDa serine/threonine protein kinase that is involved in cytoskeleton organization, cell growth, and cell survival [113,218]. mTOR along with Akt can be necessary to prevent injury in inflammatory cells [79,219] and prevent apoptotic death in dopaminergic neurons during oxidative stress [220]. mTOR also requires Akt to protect endothelial cells against apoptosis [221] and to prevent the activation of “pro-apoptotic” forkhead transcription factors [68,221]. mTOR controls apoptotic cell death through its downstream signaling pathways such as p70 ribosomal S6 kinase (p70S6K) and Bad. Phosphorylation of Bad leads to its dissociation from Bcl-2/Bcl-x_L_ and increases Bad binding to the cytoplasmic docking protein 14-3-3. Activation of p70S6K also can result in the phosphorylation of Bad, such as in astrocytes, to limit apoptotic cell injury [222]. Activation of mTOR and p70S6K may also decrease apoptosis through pathways that can increase “anti-apoptotic” Bcl-2/Bcl-x_L_ expression [222]. However, under some circumstances such as chronic neurodegenerative disorders, inhibition of mTOR may be more effective than activation of this pathway to prevent cell injury. In Alzheimer’s disease, studies have shown that post-mitotic neurons that attempt to enter the cell cycle cannot replicate and succumb to apoptosis [223,224]. In some experimental models of Alzheimer’s disease, neurons can be prevented from entering the cell cycle during the inhibition of mTOR and thus are protected from apoptosis [111,225,226]. In addition, inhibition of mTOR in murine models of Alzheimer’s disease can improve memory and reduce Aβ levels [227]. In contrast, some studies indicate that some level of mTOR activation may be required for neuroprotection. Blockade of mTOR signaling can impair long-term potentiation and synaptic plasticity in models of Alzheimer’s disease [228]. In addition, activation of mTOR and p70S6K has been shown to prevent cell death during Aβ exposure in microglia [79]. Microglia are necessary for Aβ sequestration to prevent toxicity of Aβ exposure. Other work also suggests that mTOR activity is necessary for neurite growth. Reduced mTOR activity leads to inhibition of neuronal growth, neuronal atrophy, and neuronal apoptosis [229]. Activation of mTOR in conjunction with Akt also can increase recovery from cervical spinal cord injury in rats [230].

EPO has recently been demonstrated to require mTOR activity for a variety of biological functions (Figure 1). EPO relies upon mTOR signaling for the neuronal differentiation of post-mortem neural precursors [231]. Retinal progenitor cells have been shown to be resistant to hypoxia when exposed to EPO that leads to mTOR and p70S6K activation [232]. EPO controlled bone homeostasis with osteoblastogenesis and osteoclastogenesis is dependent upon mTOR activation [233]. EPO through *wingless* signaling can activate mTOR to block apoptotic cell death in inflammatory cells [78]. In cell models of Alzheimer’s disease, Aβ degeneration of microglia is limited by EPO through combined activation of PI 3-K and mTOR pathways [79].

## 6. Conclusions and Future Perspectives

Treatments with EPO offer a number of exciting avenues to develop novel therapeutic strategies for several neurodegenerative disorders that presently lack effective treatments to either prevent or curb the devastating degree of disability that can ensue with diseases of the nervous system. In some scenarios, EPO may also function as a biomarker for disease onset and progression. For example, increased levels of EPO in the fetal plasma and amniotic fluid during gestation may serve as a biomarker of intrauterine hypoxia [45]. In addition, raised EPO serum levels appear to correlate with increased mortality in renal transplant recipients [234], suggesting that the production of EPO may be an attempt to offset toxic cellular events. EPO is present in the nervous, vascular, and immune systems that can each impact the course of neurodegenerative disorders. EPO offers robust neuroprotection in these systems against oxidative stress and apoptotic cell death.

Although EPO can affect multiple cellular pathways, new work has identified pathways that are vital for the cytoprotective capacity of EPO during oxidative stress and can impact disorders such as Alzheimer’s disease, Parkinson’s disease, amyotrophic lateral sclerosis, retinal injury, stroke, and inflammation in the nervous system. EPO relies upon *wingless* signaling with Wnt1 and the closely integrated downstream pathways of PI 3-K, Akt and mTOR. These pathways can tightly regulate the apoptotic cascade to control β-catenin, GSK-3β, mitochondrial permeability, cytochrome c release, and caspase activation.

Yet, use of EPO is not without concerns. The FDA has issued a public health advisory for erythropoiesis-stimulating agents (ESAs) that includes EPO, notifying physicians and patients of complications with ESAs that include increased rate of tumor growth and death in patients with cancer as well as blood clots, strokes, heart failure, and heart attacks in patients with chronic kidney failure when ESAs are administered to maintain hemoglobin levels greater than 12 g/dL. EPO, as a known growth factor, has been associated with tumorigenesis that may complicate administration of EPO in cancer patients suffering from anemia [235–237]. EPO treatment also may require careful modulation and in some cases, more is not better. For example, excessive over-expression of EPO may abolish any protective effects [44] and may lead to thrombotic injury [88,238]. In some clinical conditions, EPO may be contraindicated such as during severe hypertension since EPO may raise mean arterial blood pressure [11,239,240]. In an effort to limit some of these disadvantages of EPO, analogues of EPO are also under consideration. For example, asialoerythropoietin is absent of erythrogenic properties and can reduce myocardial fibrosis, inflammation, and oxidative stress in murine models of heart failure without affecting red blood cell production [86,241]. Carbamylated EPO, also without erythrogenic effects, has been shown to be neuroprotective in animal models of spinal cord injury [242]. In addition, functional agonists of the EPOR are under development for neurodegeneration and neuroprotection. However, in some cases, analogues of EPO may not offer cytoprotection [243] or neuroprotection [244], a result that may reflect low affinity binding to the EPOR. Recent studies have been carried out to improve signaling at the EPOR utilizing peptides that can specifically bind to the EPOR and have been shown to promote the survival of hippocampal and cerebellar neurons following injury with kainate or potassium chloride [147].

Given the concerns regarding EPO, identification of novel cellular pathways governed by EPO may be essential for the development of safe and effective therapeutic strategies for neurodegenerative disorders. However, understanding the complexities of these pathways will be equally important. Although activation of Wnt signaling pathways through EPO have been demonstrated to be cytoprotective and block neurodegeneration, activation of Wnt signaling in conjunction with Akt may contribute to nervous system tumors [189,245,246]. As a result, other targets for consideration that may involve the EPO-*wingless* pathway may be necessary for future consideration to foster neuronal protection. For example, recent studies show that Wnt1 inducible signaling pathway protein 1 (WISP1), a downstream target of Wnt signaling, also is neuroprotective and may represent a new approach for neurodegenerative disorders. WISP1 may modulate aging of vascular cells [181] and is protective in primary neuronal cells [193,194]. WISP1 can block GSK-3β activity in cells [193,247]. During the inhibition of GSK-3β, β-catenin is not phosphorylated, ubiquinated, or degraded and can translocate to the nucleus to prevent cellular apoptosis [77,186]. WISP1 through a PI 3-K mediated pathway promotes the translocation of β-catenin from the cytoplasm of neurons to the nucleus that can allow for the transcription and eventual translation of pathways that can limit apoptotic cell death [194]. Other studies have suggested that activation and phosphorylation of Akt and mTOR may be associated with the progression of Alzheimer’s disease [248]. Inhibition rather than activation of mTOR may be required for the treatment of epilepsy [249]. In addition, excessive mTOR activity may contribute to dyskinesias in Parkinson’s disease patients [250]. Future studies that can elucidate the intricate biological function and relationship of EPO and the pathways of Wnt, PI 3-K, Akt, and mTOR should open new directions for EPO and its signaling pathways as clinically effective strategies for the nervous system.
